# Research on the reproductive biological characteristics of *Amomum villosum* Lour. and *Amomum longiligulare* T. L. Wu

**DOI:** 10.1371/journal.pone.0250335

**Published:** 2021-08-19

**Authors:** Ruipei Yang, Jie Wang, Wei Gao, Ye Jiang, Jing Su, Dongmei Sun, Guozhen He

**Affiliations:** 1 Shenzhen Key Laboratory of Hospital Chinese Medicine Preparation, Shenzhen Traditional Chinese Medicine Hospital, The Fourth Clinical Medical College of Guangzhou University of Chinese Medicine, Shenzhen, Guangdong, China; 2 Fifth Clinical Medical School, Guangzhou University of Chinese Medicine, Guangzhou, Guangdong, China; 3 Guangdong Second Traditional Chinese Medicine Hospital (Guangdong Province Engineering Technology Research Institute of T. C. M), Guangzhou, Guangdong, China; 4 Guangdong Provincial Key Laboratory of Research and Development in Traditional Chinese Medicine, Guangzhou, Guangdong, China; 5 Shenzhen People’s Hospital, Shenzhen, Guangdong, China; 6 Guangdong Yifang Pharmaceutical Co., Ltd. Foshan, Guangdong, China; 7 College of Chinese Traditional Medicine, Guangzhou University of Chinese Medicine, Guangzhou, Guangdong, China; 8 Yangchun Field Test and Demonstration of Amomum Villosum, Yangchun, China; Universita degli Studi della Calabria Dipartimento di Biologia Ecologia e Scienze della Terra, ITALY

## Abstract

**Objective:**

To explore the influence of biological characteristics on the yield of *Amomum villosum* Lour. and *Amomum longiligulare* T. L. Wu, to find an effective pollen viability evaluation method and storage method to solve the problem of the low yield of *Amomum* plants.

**Methods:**

Five germplasm of *Amomum* plants were used to investigate the effects of the phenological phase, pollen viability, and stigma receptivity on natural and artificial fruit set.

**Results:**

*Amomum longiligulare* T. L. Wu showed late flowering, and its natural pollination rate is higher than that of *Amomum villosum* Lour. In all germplasm, the artificial pollination rate and fruit setting rate are more than 3 times higher than that under natural conditions. Fruits begin to drop seven days after successful pollination, and the fruit drop is basically stable after one month. The hybridization verification showed that TTC method was simpler and more accurate than in vitro germination method. Optimal storage conditions for pollen are 4°C and high humidity. After 36 h of storage, pollen can still be used for artificial cross-pollination or as hybrid parents.

**Conclusion:**

The special biological characteristics are the fundamental reason for the low natural pollination rate of *Amomum* plants. The accurate measurement method of *Amomum* plants pollen is the TTC method, and storage at 4°C and high humidity can increase the yield, which was six times that of the natural yield.

## Introduction

*Amomum villosum* Lour. and *Amomum longiligulare* T. L. Wu (genus *Amomum*, family Zingiberaceae) are two of the basic species of the traditional Chinese medicine *amomi fructus*(also called Sharen), which are cultivated as medicinal plants in the Guangdong, Guangxi, Hainan and Yunnan provinces of China with great pharmacology effect [1]. Amomi fructus/Fructus amomi (FA)is commonly used in the clinical practice of traditional Chinese medicine and can also be used in food, drink, health products and seasonings [2–4]. It is estimated that the market demand of the FA industry is more than $1.4 billion [5]. In recent years, the average price of FA as “*Daodi* herb” [6] from Production Areas in Guangdong exceeded $150 per kilogram [5]. The weight of one hundred dried ripe fruits of FA are almost 50g [7], and the values are up to approximately $0.15 of a single FA. According to 2.25×10^5^ inflorescences per hectare, each inflorescence 10.6 florets [5], if all results can be achieved, the output value would up to $357,750.

However, the supply for FA is short of the demand due to its low yield per hectare; the main reasons being the low natural pollination rate and the high fruit drop rate [8]. Fruit shedding is a physiological phenomenon of plants caused by shortage of nutrients [9]. In most parts of China, artificial pollination is the main planting method because lack of pollinators or lower effectiveness of the pollinators [10, 11]. At present, the germplasm types of *A*. *villosum* Lour. (include ‘*A*. *villosum* cv. Changguo’, ‘*A*. *villosum* cv. Yuanguo’, ‘*A*. *villosum* cv. Jinqi’and ‘*A*. *villosum* cv. Zhonghua’ [12]) and *A*. *longiligulare* are the major Citrus species cultivated and used in the China [7]. The effect of the five *Amomum* plant cultivars choice on yield and the reasons for the low natural pollination rate of them were less clear. Some have argued that the special flower structure makes it difficult to self-pollination, and the active time of insects that can effectively pollinate is inconsistent with the flowering period, which is not very helpful in promoting pollination [4]. Nevertheless, pollinating insects are important factors in the formation of *Amomum* plants, but not the only conditions, effective pollination is a precondition for the fruit set of most plants. At present, the yield of FA in Yunnan Province is still very low in the production areas where do not lack insect pollinators [10].

Pollen vitality, stigma receptivity, the law of pollen-pistil interaction and other pollen biology information that are tightly related to productivity have received little attention [13]. To date, researches on the simultaneous determination of pollen vitality of *A*. *villosum* Lour and *A*. *villosum* were not reported. We speculate that the low natural fruit set may be a multifactorial process, which stimulated our interest to solve low yield by understanding the biological characteristics of multiple germplasm *Amomum* plants, combining the phenological period, pollen vitality, and stigma acceptability.

In the present study, five germplasm of *Amomum* plants were used to investigate the effects of the phenological phase, pollen viability, and stigma receptivity on natural and artificial fruit set. The objectives of the study were to investigate: 1. To assess the effect of 5 cultivars at different flowering stages and artificial pollination on fruit set. 2. Evaluate and improve pollen viability tests and the law of pollen vitality and stigma acceptability over time. 3. To identify factors influencing viability of pollen and the pollination rate of *Amomum* plants. 4. To confirm the yield effect of artificial pollination on various *Amomum* plants under farmer’s field conditions.

## Materials and methods

### Plant material

Five herbaceous *Amomum* germplasms were selected according to a previous investigation: ‘*Amomum longiligulare* T.L.Wu’, ‘*Amomum villosum* cv. Changguo’, ‘*Amomum villosum* cv. Yuanguo’, ‘*Amomum villosum* cv. Zhonghua’, and ‘*Amomum villosum* cv. Jinqiu’.

### Location and climate of study sites

This study was carried out from April 2015 to July 2017, in the Shizhen Mountain and Chinese Medicine Resources Laboratory of Guangzhou University of Traditional Chinese Medicine (113° 14ʹ ~113° 34ʹ N; 22° 45ʹ~23° 05ʹ E, at an altitude of 3–18 m), Guangzhou (GZ), and the center of a large *Amomum*-growing field (111° 44.5ʹ N; 22° 12.7ʹ E, at an altitude of 32–43.6 m), Yangchun (YC), Guangdong province, Southwest China. The annual average precipitation is 1673.0 mm (GZ), 2380 mm (YC)and annual mean temperature reaches 22.5°C (GZ), 22.0°C (YC) in the study site. Rainfall in the study areas are seasonal and occurs from April to June. Humidity ranges from 67 to 85% across all sites. YC were identified for the study which have been considered as the main large *Amomum* growing areas in China.

### Observations of flowering dynamics

Typical *Amomum* plant flowers were selected to observe the biological characteristics of flowering and the dynamic rules of flower opening from the bud stage to the final flowering stage.

### Observations of pollination and fruit dropping

Natural pollination was carried out by bagging a single inflorescence (in a 6 cm×10 cm sulfuric acid paper bag) before the flower opened. Artificial pollination was carried out by giving pollen from the same flower immediately after flowering. Every seven days after pollination, the samples were observed continuously for one month to record the fruiting rate and fruit drop rate. The fruit set (number of fruits/ treated flowers per inflorescence) and Fruit drop ((the max number of fruits—the final fruits number at harvest / the max number of fruits) [14].

#### Hand-pollination experiment

First of all, use the thumb and ring finger of one hand to clamp the lower part of the corolla. Secondly, the middle finger and index finger clamp the major lip, and then use the index finger (or bamboo slice) of the other hand to insert between the major lip and the stamen anther. Finally, apply pollen to the stigma (S1 Fig).

### Pollinator observations

In 2017, Flower visitors and their behavior were observed from April to July at two populations of *Amomum* plants. At each population, we set two 2 × 2 m plots. Observations were conducted continuously from 08:00 to 19:00, to determine the visiting the types, and behaviors of pollinators. We recorded a plant visitor as a pollinator if it touched the stigmas or anthers of the flower during its visit by camera. At the end of the visit, a minimum of three individuals flowers per group was evaluated under a Stereoscopic.

### Pollen collection and storage

During the flowering period, pollen was collected between 7:00 and 8:00 a.m. on a sunny morning, which guaranteed that the flowers grew vigorously on the same plot. The samples were placed in a 1.5 mL centrifuge tube and divided into four storage conditions: (1) Freshly cut stigmas: the pollen was collected randomly every 3 h, and the viability was immediately measured; (2) Room temperature and high humidity: the flower was quickly placed on a pad with a layer of wet filter paper in a culture dish, sealed at room temperature (approximately 28°C), and placed in constant temperature storage; (3) Refrigerated at high humidity: the flowers were quickly placed on a pad with a layer of wet filter paper in a culture dish, then placed in the refrigerator (4°C); (4) Dried at low temperature: the anthers and the pollen were packed in hermetic bags with silica gel after the anthers were desiccated and the pollen was placed in the freezer (-20°C). The germplasm pollen samples were stored for ten different time intervals, viz. 0 h, 3 h, 6 h, 9 h, 12 h, 24 h, 27 h, 30 h, 33 h, and 36 h.

### Pollen germination

The prepared culture medium (formula: 3% sucrose+10 ppm boric acid) was added to the groove of a concave glass slide [15], then a small amount of the pollen to be measured was evenly sprinkled in the culture solution; the glass slide was then placed into a culture dish covered with moist moisturizing filter paper and cultured at normal temperature, with 3 repetitions for each combination. Pollen germination was evaluated under a microscope after 5 h. Images were randomly captured of three nonoverlapping fields of the fixed pollens The pollen grains were considered to be germinated when the pollen tube was equal to or greater than the diameter after being cultured for five hours at 25 °C.

### Viability test

For the 2,3,5-triphenyltetrazolium chloride (TTC) staining test, 0.05 g of TTC was dissolved in 100 mL phosphate buffer (pH 7.17). Several minutes after staining, the stained pollen grains were scored as “viable”, while abnormally sized and unstained pollen were scored as “nonviable” [13, 16].

Pollen vitality = viable pollen grains / total observed pollen grains x 100%.

For this method, observation was carried out under a 100× magnification light microscope, and at least 30 pollen grains should be randomly counted in each field.

### Stigma receptivity test

Stigma receptivity was determined by the benzidine-hydrogen peroxide method [17, 18]. During the flowering period in *Amomum* plants, a single plot was randomly selected for flowering on the same day. The flowers were collected at different times after flowering began at 7:00 in the morning, and the stigma was placed on a concave slide and immersed in the 1% benzidine-hydrogen peroxide reaction solution (the aniline:3% hydrogen peroxide: water ratio was 4:11:22, by volume of the depression). If the column head was receptive (presenting peroxidase activity), the reaction solution around the column head appeared blue with a large number of bubbles.

### Verification by hybridization

When the flowering season occurred, ‘*A*. *villosum* cv. Yuanguo’ was selected as the female parent and ‘*A*. longiligulare’ was selected as the male parent. Pollen stored at 4 °C and high humidity and fresh pollen stored for approximately 36 h were chosen for the hybridization test.

### Statistical analysis

The data for the percentage of pollen germination on the stigma, fruit set, and fertilization were expressed as the mean ± *SD*. The least significant difference was determined using the SPSS 24.0 software. Differences between groups were determined by the *Games-Howell* or *SNK test* at *P*<0.05.

## Results

### Biological characteristics of flowering

From mid-May to early July, ‘*A*. *villosum cv*. Changguo’ and ‘*A*. *villosum cv*. Yangguo’ flowered first, followed by ‘*A*. *villosum cv*. Zhonghua’, ‘*A*. *villosum cv*. Jinqiu’ and ‘*A*. *longiligulare*’ in order. The flowering of ‘*A*. *villosum cv*. Changguo’ and ‘*A*. *villosum cv*. Yangguo’ in 2017 occurred on 14/15 May, which was 7 ~ 9 days later than that of ‘*A*. *villosum cv*. Zhonghua’ and ‘*A*. *villosum cv*. Jinqiu’ and 22 ~ 23 days later than that of ‘*A*. *longiligulare*.’ The florescence of ‘*A*. *villosum cv*. Zhonghua’ and ‘*A*. *villosum cv*. Jinqiu’ was 34 ~ 35 days; that of ‘*A*. *villosum cv*. Changguo’ and ‘*A*. *villosum cv*. Yangguo’ was 26 days; and that of ‘*A*. *longiligulare*’ was only 30 days. ‘*A*. *villosum cv*. Zhonghua’ and ‘*A*. *villosum cv*. Jinqiu’ had the longest blooming period of 24 days, while that of ‘*A*. *villosum cv*. Changguo’ and ‘*A*. *villosum cv*. Yangguo’ was less than 10 days and that of ‘*A*. *longiligulare*’ was in the middle, at approximately18 days.

**Fig 1:**
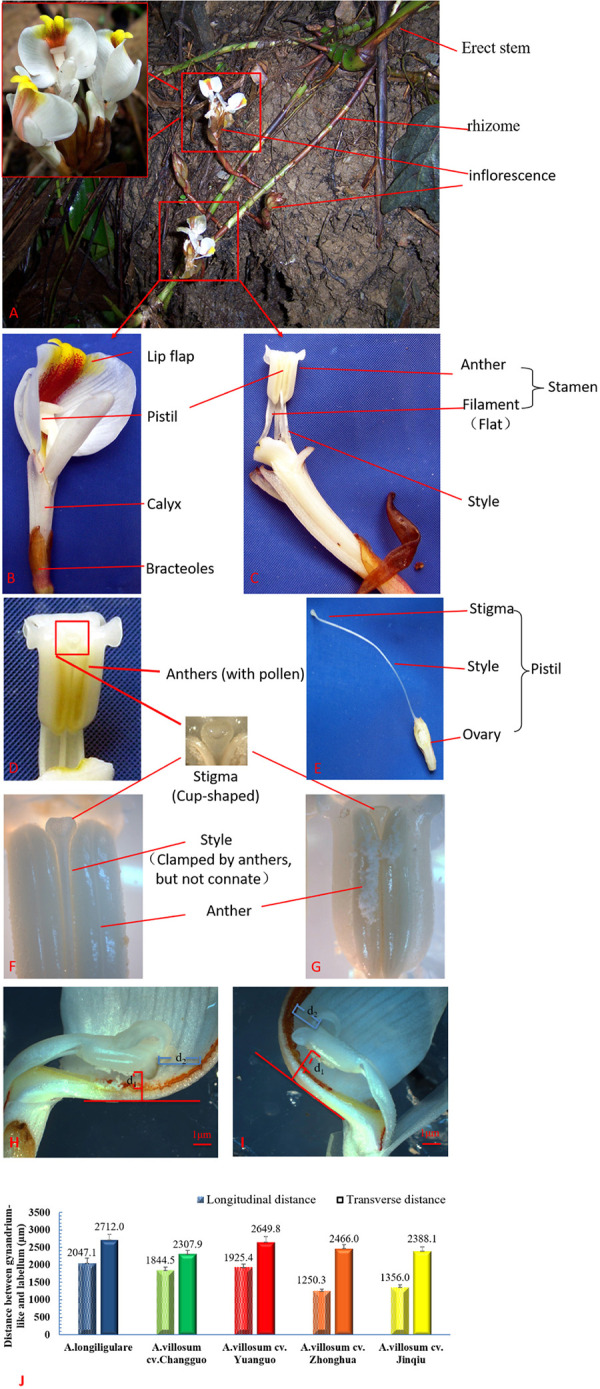
Anatomy of the flower-shaped structure of Amomum villosum. (A) The flowers on the inflorescence gradually open from bottom to top, (B) Whole flower, (C) Stamen, (D) Stigma and pollen sac (the position is higher than the anther, therefore, it is difficult for pollen to enter the cup), (E) Pistil, (F) Unexploded anther sac, (G) Pollen sac that is longitudinally split with the pollen grains outside, Illustration of the method for measuring longitudinal (H) and transverse (I) distances between gynandrium-like and labellum, (k) Histogram of distances between gynandrium-like and labellum of five Amomum germplasms (n = 9).

Through dynamic observation, the inflorescences of the five kinds of *Amomum* germplasm were all extracted from the rhizomes, the inflorescences were racemes, and the florets gradually opened from bottom to top. There was no obvious difference in the floret morphology. The floral organ had a special structure. The style was sandwiched between the left and right pairs of pollen sacs. The stigma was higher than the anther and had an upward opening. The pistils and stamens were half-enclosed in the lip, and the cleft of the pollen sacs was attached to the lip (Fig 1A–1G). The Longitudinal distance between gynandrium-like and labellum less than 2μm, and the Transverse distance between gynandrium-like and labellum less than 3μm (Fig 1H–1J). ‘*A*. *longiligulare*.’ usually had 6–9 florets in each inflorescence, with an average of 7.28±0.83, and the florescence of a single flower was 1.5 days. However, the inflorescence of the other four types of Amomum had an average of 9–10 florets, and the opening time was usually one day (Table 1).

**Table 1:** Comparison of flowering phenology of 5 herbaceous *Amomum* germplasms.

Year	Flowering period	*A*. *longiligulare*	*A*. *villosum cv*. Changguo	*A*. *villosum cv*. Yunguo	*A*. *villosum cv*. Zhonghua	*A*. *villosum cv*. Jinqiu
2017	First flowering (Month/Day)	6/2	5/15	5/14	5/23	5/22
	Peak flowering (Month/Day)	6/6-6/24	5/21-5/30	5/21-5/30	5/27-6/20	5/27-6/20
	Late flowering (Month/Day)	6/24-7/3	5/30-6/5	5/30-6/4	6/20-6/26	6/20-6/26
Number of flowers per inflorescence		7.28±0.83a	9.06±1.86b	10.00±1.94b	9.27±1.99b	9.89±1.88b

Means within a germplasm followed by the same letter do not differ at *P* < 0.05 (*SNK test*).

### Fruit setting rate and fruit drop rate

**Fig 2:**
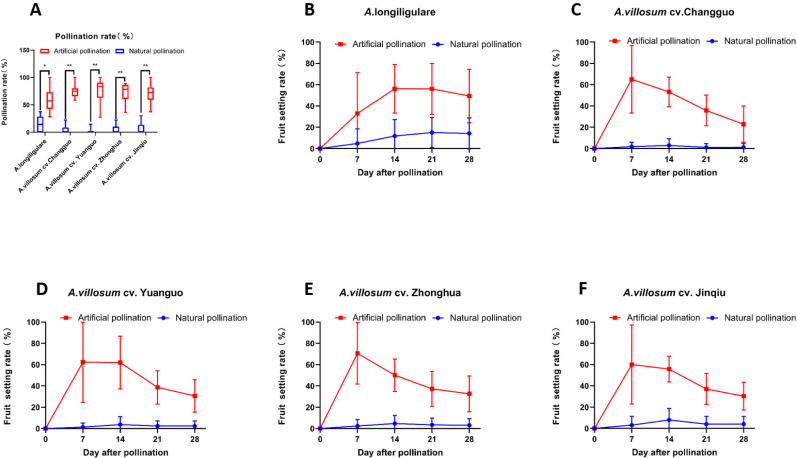
Effects of different pollination methods. (A) Different Pollination rate between Natural pollination and Artificial pollination, (B-F) Change with time after imposition of Pollinations in fruit setting rate of 5 *Amomum* germplasms between Natural pollination and Artificial pollination. n = 18 per group. Data are presented as mean ± SD. *P<0.05, **P<0.01.

Under natural pollination conditions, the highest pollination success rate, number of results, and fruit setting rate were, respectively, 14.91 (%), 1.06 (pieces), and 14.24 (%) for ‘*A*. *longiligulare*’, and 3.45 (%), 0.11 (pieces), and 1.23 (%) for ‘*A*. *villosum cv*. Changguo’ (the lowest rate). There was no difference in the fruit drop rate of the five Amomum varieties. Under artificial pollination conditions, the resulting number and fruit setting rate of ‘*A*. *longiligulare*’ were the highest at 3.67 (pieces) and 49.38 (%), respectively, while the pollination success rate and fruit drop rate were the lowest at 56.07 (%) and 20.65 (%), respectively. The artificial pollination success rate and fruit setting rate of the five *Amomum* germplasms were at least 3 times higher than those under natural conditions (Fig 2A). However, there was no significant difference in fruit drop rate between the two conditions (Table 2). Results show that fruit drop began to emerge seven days after successful pollination (starting from 14 days after natural pollination), and the fruit drop rate is basically stable after 1 month of fruit set (Fig 2B–2F).

**Table 2:** Effects of different pollination methods on fruit setting rate and fruit dropping rate of 5 amomum germplasms in response to pollination.

Cultivar	Natural pollination			Artificial pollination		
	Pollination rate (%)	Fruit setting rate (%)	Fruit drop rate (%)	Pollination rate (%)	Fruit setting rate (%)	Fruit drop rate (%)
*A*. *longiligulare*	14.91±12.74a^a^	14.24±14.51a	15.38±37.55	56.07±24.06A^b^	49.38±25.09A	20.65±31.39a
*A*. *villosum cv*. Changguo	3.45±6.36b	1.23±3.54b	60.00±54.77	75.54±11.98B	22.88±17.01B	71.55±19.33b
*A*. *villosum cv*. Yuanguo	2.38±4.77b	1.78±4.17b	30.00±44.72	76.46±20.74B	30.64±15.23B	58.47±16.32b
*A*. *villosum cv*. *Zhonghua*	4.72±7.68ab	3.02±6.31b	41.67±49.16	71.81±17.44B	32.61±16.83B	59.19±19.28b
*A*. *villosum cv*. Jinqiu	7.89±10.78ab	4.07±7.12ab	47.92±46.66	70.77±17.43B	30.31±13.05B	59.36±16.51b

^a^Mean values (n = 18) followed by different lowercase letters in each column indicate significant differences at *P*< 0.05 by the *SNK test*.

^b^Mean values (n = 18) followed by different uppercase letters in each column indicate significant differences at *P*< 0.05 by the *Games-Howell test*.

### Viability of fresh pollen

We found the pollen viability observed by TTC staining was higher than that from the in vitro germination test. The vitality of all varieties assessed by the TTC staining method exceeded 94.00%, while only one variety tested by in vitro germination exceeded 80.00% (Table 3). In vitro germination confirmed that the maximum germination rate of fresh pollen appeared in ‘*A*. *longiligulare*’ (83.59%), and the minimum pollen vitality appeared in ‘*A*. *villosum cv*. Changguo’ (55.06%) (Table 3).

**Table 3:** Viability of fresh pollens was confirmed by the TTC staining method and in vitro germination in herbaceous *Amomum* cultivars.

Cultivar	Pollen viability test (%)	
	TTC staining method	In vitro germination
*A*. *longiligulare*	95.57±1.63	83.59±1.44a^a^
*A*. *villosum cv*.Changguo	94.25±1.91	55.06±8.42b
*A*. *villosum cv*. Yuanguo	94.88±1.50	60.39±3.94b
*A*. *villosum cv*. Zhonghua	95.27±1.58	78.03±1.56ac
*A*. *villosum cv*. Jinqiu	95.51±1.85	75.58±2.92ac

The results are the mean ±*SE* (n = 3).

^a^Mean values (n = 3) followed by different lowercase letters in each column indicate significant differences at *P*< 0.05 by the *SNK test*.

### Pollen longevity at different storage temperatures

**Fig 3:**
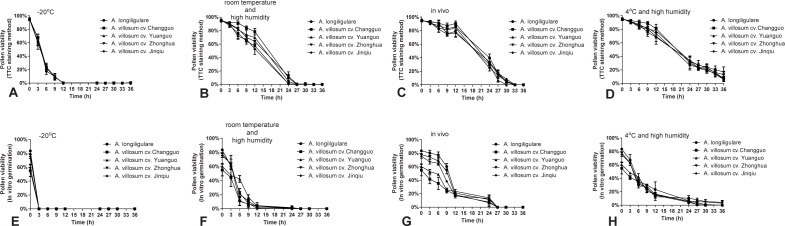
Pollen viability of the five cultivars ‘*A*. longiligulare’, ‘*A villosum cv*. Changguo’, *A*. *villosum cv*. Yuanguo’, ‘*A*. *villosum cv*. Zhonghua’ and ‘*A*. *villosum cv*. Jinqiu’, stored in vivo at 4 °C and high humidity and at room temperature as tested by two methods. TTC staining method (A–C) and in vitro germination test (D–F).

The TTC staining method and in vitro pollen germination test showed that the pollen activity decreased fastest under the dry and low temperature (-20°C) condition. The TTC staining showed the viability of all varieties decreased to 0.00% at 12 h (Fig 3A), while the in vitro germination showed that all pollen was inactive (0.00%) after 3 h (Fig 3E).

The *Amomum* germplasms ‘*A*. *villosum cv*. Changguo’ (1.52%), ‘*A*. *villosum cv*. Yuanguo’ (1.33%), ‘*A*. *villosum cv*. Zhonghua’ (0.57%) and ‘*A*. *villosum cv*. Jinqiu’ (0.76%) (Fig 3B) could still survive after being stored at room temperature and high humidity for 24 hours, while the pollen viability of ‘*A*. *longiligulare*’ dropped to zero (in vitro germination). After storage at room temperature and high humidity for 12 h, the pollen viability of ‘*A*. *longiligulare*’ (Fig 3B) decreased to 0.57% (in vitro germination); however, the pollen viability measured by the TTC staining method was 57.49% (Fig 3F).

The TTC staining and in vitro germination tests of pollen viability showed that the pollen viability percentage was significantly higher for pollen stored in vivo than that stored at room temperature and high humidity or -20°C. After 24 hours of storage in vivo (in vitro germination), the pollen from ‘*A*. *longiligulare*’ (11.37%), ‘*A*. *villosum cv*. Changguo’ (6.66%), ‘*A*. *villosum cv*. Yuanguo’ (13.83%)’, ‘*A*. *villosum cv*. Zhonghua’ (7.65%) and ‘*A*. *villosum cv*. Jinqiu’ (12.95%) were alive (Fig 3G).

The pollen viability of all Amomum varieties (Fig 3G) decreased to 0.00% after 27 h of storage in vivo (in vitro germination); however, the TTC staining showed that they still maintained high survival rates of 16.88%, 15.70%, 6.70%, 13.97% and 17.26% (Fig 3C). The pollen viability was markedly lowered after 24 h.

The TTC staining and in vitro germination results confirmed that the pollen viability of all Amomum varieties was the highest at 4°C and high humidity in the first 36 h of storage, compared with other storage temperatures. At all observation times, the pollen viability at 4 °C and high humidity showed a similar trend when comparing the five herbaceous Amomum varieties. The pollen viability decreased significantly over a period of up to 24 h, similar to when it was stored in vivo. When tested by the in vitro germination method, the germination rate decreased slowly from 12 h to 24 h of storage (Fig 3D and 3H). After storage at 4°C and high humidity for 36 h, all five herbaceous Amomum germplasms could still survive (Fig 3D).

In general, except at -20°C, the TTC staining showed that the decline rate of pollen viability under the three storage temperatures was similar, and the slowest decline rate occurred under the conditions of 4°C and high humidity.

### The effect of time on the stigma receptivity

**Fig 4:**
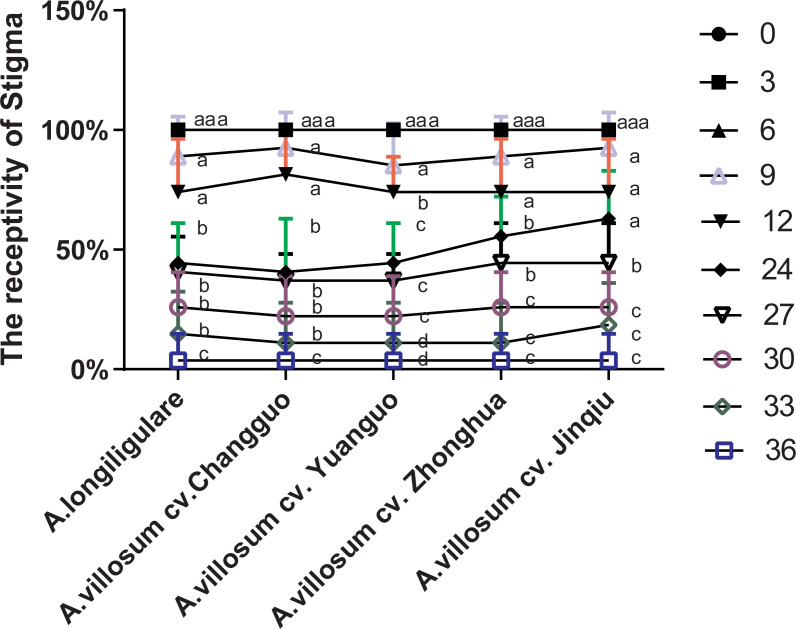
Stigma receptivity of the 5 herbaceous *Amomum* varieties. n = 3 per group. Data are presented as mean ± SD. Means within a cultivar followed by the same letters do not differ at *P* < 0.05 (*SNK test*).

The stigma receptivity was different in the different germplasms, and the stigma receptivity of the fresh flowers was directly related to the time after flowering. Higher stigma receptivity was measured at 24 h for all of the studied germplasms (Fig 4). The stigma receptivity was the most potent on the day of flowering and achieved good peroxidase activity. After 19:00 on the 2nd day, the stigma darkened and the peroxidase activity weakened to none.

### Hybridization experiment

**Fig 5:**
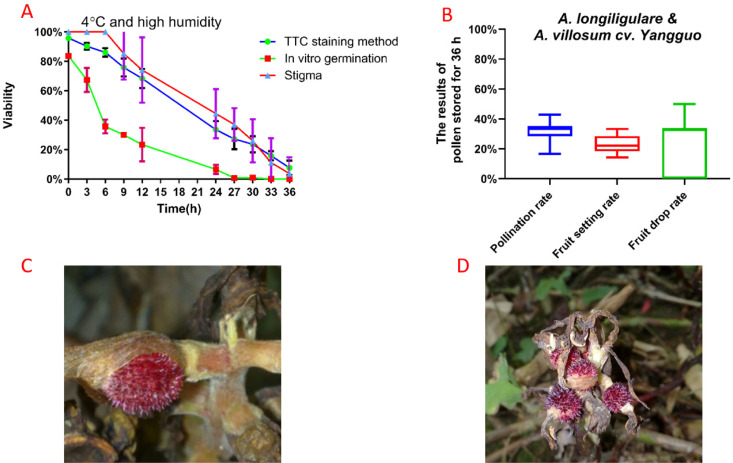
Verification by hybridization. (A)The pollen viability of ‘*A*. *longiligulare*’ stored at 4 °C and high humidity was tested by in vitro germination and the TTC staining method, (B)The results of pollen stored for 36 h, (C-D) A model for fruit set in the treatment of Hybridization in two *Amomum* plants.

Experiments show that ‘*A*. *longiligulare*’ had a high fruit setting rate (Table 2). The yield of ‘*A*. *villosum cv*. Yangguo’ was extremely low (Table 2), and the fruit did not develop well without artificial pollination. However, we found that even if the viability of ‘*A*. *longiligulare*’ pollen also decreased to 0.00% (in vitro germination) and 7.66% (TTC staining method), the fruit setting rate from the pollen of the offspring from the female parent ‘*A*. *villosum cv*. Yangguo’ and male parent ‘*A*. *longiligulare*’ was still high for both pollen stored for 36 h and fresh pollen (Fig 5B). Before 30 h, the stigma receptivity of ‘*A*. *longiligulare*’ was slightly higher than its pollen viability, and the most vigorous time for both was 6 h after flowering (> 80.00%) (Fig 5A, S2 Fig).

### Main flower-visiting insects and flower-visiting behavior

**Fig 6:**
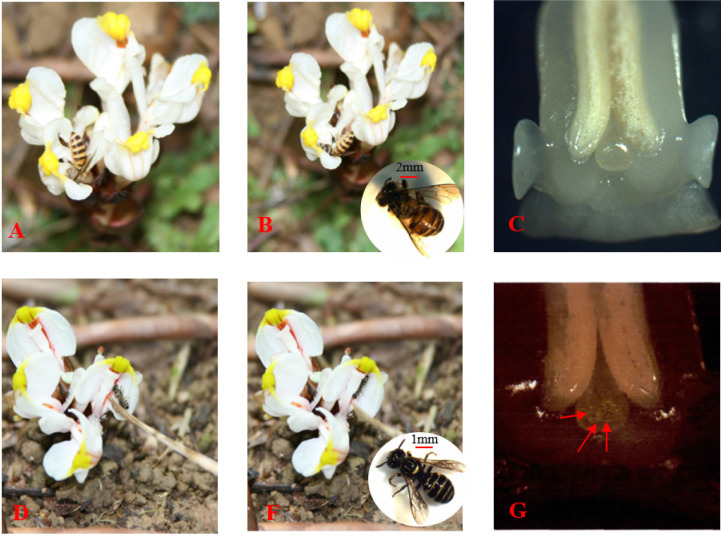
Foraging dynamics of honey bees (A, B) and *Nomia strigata* Smith (D, F), pollination effectiveness of honey bees (C) and *Nomia strigata* Smith (G) were observed using a stereomicroscope.

Among the two species of visitors we observed, different insects behaved diversely when visiting. *Apis (Siamatapis) cerana* Fabricius (honey bees) (Fig 6A and 6B) were the most frequent visitors and *Nomia strigata* Smith (Fig 6D and 6E) were the most effective visitors of *Amomum* plants. Honey bees enter the flowers through the stigma-anther column from either side and during this movement, while nectar foraging, they move faster and last about 5 s. We observed that pollens were not found on the stigma cup of the flowers under a stereomicroscope (Fig 6C). Sporadic appearance of *Nomia strigata* Smith were observed at the early July. The size of the *Nomia strigata* Smith is only half the size of the honey bees, and them acted differently for nectar collection. It visits the flowers from the upper part of the gap between gynandrium-like and labellum. The head is at the lower end of the gynandrium-like, the tail is close to the stigma, and the abdomen is in contact with a lot of pollen. Time spent in flowers during nectar extraction/foraging was higher, sometimes up to about one minute. We observed that pollens were released on the stigma cup of the flowers during foraging under a stereomicroscope ((Fig 6G).

## Discussion

Due to their special structure and lack of pollinators or lower effectiveness of the pollinators, the yield of *Amomum villosum* and *A*. *longiligulare* could not maintain by natural pollination. The present study has demonstrated that the late-flowering varieties of the five germplasms of *Amomum* plants have higher natural pollination rate and lower fruit drop rate, but the success rate of hand pollination (artificial pollination) of all varieties is more than 3 times that of natural pollination. Further, there is no difference in fruit drop rate and thus increasing fruit set. Secondly, the study showed that TTC method can accurately detect pollen viability. And yield is impaired due to reduced pollen viability, and stigma receptivity, while, thirdly, it showed that the optimal storage conditions for pollen are 4°C and high humidity, which can still be used for hybridization or artificial pollination after being stored for 36 h.

We found that, late flowering varieties had the highest fruiting rate in the natural pollination test group, but it was still less than one-third of artificial pollination. The fruit setting rate of artificial pollination is more than 3 times that of natural pollination, but there is no difference in fruit drop rate. After artificial pollination of the five germplasms of *Amomum* plants, more than 50% of fruits were enlarged. Fruit enlargement occurs in less than 15% of flowers in natural pollination. This may correlate with the biological function of its flowers. The anatomical structure of the flower was examined from several series of transverse and longitudinal sections in the floral buds at different stages of growth. We found them have a special structure, where the pistil stigma is higher than the stamen anther, and the style is embedded in the gap between two anther chambers. Therefore, it is difficult for pollen to fall on the stigma and self-pollinate (Fig 1). Pollen grains have spiny protuberances on their surface, making them easy to adhere to each other. Therefore, it is less likely to cross-pollinate by wind. Indeed, the distance between the stigma and the petals is narrow (Fig 1H and 1J), and there are very few insects that can effectively pollinate. This finding is different from the conclusion that both honey bees and bumble bees contribute to pollination and enhance yield of large cardamom [19]. Our previous study found that Nomia sp. are the most effective pollinating insects in Guangdong Province. Stereoscopic observation of the stigma after insect visits showed that the pollination effect of the Nomia sp. is obvious. The honey bees were pollen thieves rather than pollinators. Before the farmers conduct artificial pollination, the pollen is taken away, which brings difficulties to artificial pollination [20]. In addition, the active period of Nomia sp. is at the end of June and beginning of July in Guangdong Province, and they mostly appear in the late flowering period, which has little benefit for pollination of *Amomum villosum* [21]. This is one of the reasons for a low rate of fruit setting. Therefore, the selection and breeding of late-flowering varieties may be an important direction to solve low yield in the future.

Compared with the in vivo germination method, the TTC method is more accurate in detecting pollen viability. Both pollen viability and pollination time of stigma not long are another important reason for low yield. This study showed that survivability results with similar trends have been obtained through different detection methods, further verifying the reliability of the experiment. From hybridization experiment, it also follows that even if the viability of ‘*A*. *longiligulare*’ pollen also decreased to 0.00% (in vitro germination) and 7.66% (TTC staining method), the fruit setting rate was still high for pollen stored for 36 h (Fig 5B). Although the TTC staining method has the problem of false positives in other experiments [22], in this study, the TTC staining method can detect pollen viability more reliably and is simpler. The basic trend reflected by the in vitro germination method was similar to the TTC method; however the in vitro germination method was often restricted for various reasons, resulting in a low germination rate that was unable to reflect the true pollen vitality [23].

*Amomum* plant’s florescence is only 24–36 h, and the stigma has strong receptivity after flowering, but it only lasts for less than 24 h. As it was shown in Fig 4, the stigma began to weaken within 12 h, and such weakening was aggravated within 24 h. In this experiment, the long-term observation of *Amomum* plants showed that the fruit drop rate was stable after one month of fruit setting. The natural fruit setting rate was as low as 1.23% and as high as 14.24% (Table 2). The reason for this low natural fruit setting rate should be the short duration of pollen and stigma vitality. Perennial *Amomum* has more than 7 flowers per inflorescence (S3 Fig), and the flowers open sequentially from bottom to top. If the pollen from the same plant that blooms on the same day is collected for the artificial pollination of stigma-mature flowers, a higher fruit setting rate can be achieved. For example, the variety ‘*A*. *villosum cv*. Changguo’ had the lowest natural fruit setting rate of 1.23%, which increased to 22.88% with artificial pollination, while the fruit setting rate of ‘*A*. *longiligulare*’ increased to 49.38%. Therefore, optimizing artificial pollination is the most effective way to solve the problem of the low fruit setting rate of *Amomum* plants. To be specific, studying how to store pollen more effectively, and exploring more effective methods to evaluate pollen vitality are very important.

The findings from this study showed that the *Amomum* plant’s pollen viability was significantly influenced by low temperatures and high humidity. Compared to other storage conditions, the pollen germination rate increased at the later stages of storage after being cultured in vitro at 4°C and high humidity. Pollen vitality could be prolonged by nearly 9 hours (Fig 1C and 1D) compared to in vivo pollen through simple storage means such as at 4°C and high humidity, which is beneficial to actual artificial pollination and later crossbreeding research. However, there is no clear explanation for this difference. Factors affecting the pollen storage life during storage are not just temperature and humidity [24, 25], moisture content should also be included [26, 27]. Even if it is stored for a short period of time, the differences in temperature and relative humidity will also have critical influences on the germination and growth of pollen [25].

In addition, it has been reported that pollen grains with high early moisture content are not suitable for closed storage since they are sensitive to sealing and have a short storage life [28]. A dry environment is a common storage method for pollen, not only because it can inhibit the growth and reproduction of microorganisms, but more importantly because it can lessen water-mediated degradation reactions to help preserve pollen [29–31]. However, in our previous research, we found that the pollen vitality of *Amomum villosum* was temporarily reduced due to high temperatures and low humidity. When the temperature was reduced and the humidity was raised, again vitality increased [20]. This coincidence was different from previous researchers’ conclusions [32]. Amomum has triple karyotype pollen. Generally, it is believed that the germination rate of triple karyotype pollen is lower than that of double karyotype pollen. The outer wall of triple karyotype pollen is thin and susceptible to dryness. Triple karyotype pollen has a short life span, and its vitality is better under high humidity conditions [33]. On the other hand, the lowest activity at -20°C may be because of water crystals caused by low temperatures under insufficient drying, resulting in a loss of activity under low temperature storage [31].

At room temperature, strong respiratory and metabolic activities, severe water loss, high temperature and humidity, or a rapid decline of activity will lead to poor storage characteristics. However, under freeze-drying conditions, the metabolism will be delayed, and enzyme activity and respiration will be weakened, thus effectively slowing down the decline rate of pollen activity. JIM (2003) further noted that storing pollen under suitable conditions can prolong its life because pollen metabolism weakens under low temperature, dry and oxygen-free conditions, and pollen can keep its vitality for a long time [34]. In contrast, under high temperature, high humidity and aerobic conditions, the metabolism is more active, nutrient consumption and enzyme activity declines, and vitality is easily lost [35].

Interestingly, when the TTC method was used for examination, after 9 h, we found that the pollen stored in vivo had a survival rate of less than 12 h (Fig 3C). Most likely, the stored humidity in the field environment was too low and the temperature was too high after 6 h, which will have a negative impact on the pollen performance between 13:00 pm and 16:00 pm. However, this result was inconsistent with the previous research in cherimoya [36]. A reasonable explanation could be that under natural conditions, the pollen viability increases with the increase of temperature within a certain range, and beyond that range, the pollen viability will decrease as the temperature increases. This was consistent with the conclusions of previous scholars [32].

Finally, the correlations between field and greenhouse or laboratory evaluations needs to be considered. Since the seventies, Large scale artificially pollinated efforts have been undertaken in the main producing areas of China [11]. In addition, it is low cost, simple to perform, and requires minimal training of Farmer. From S1 Fig shows the method reported here is very straightforward and relatively simple to implement. According to this study, during artificial pollination, just scrape off the excess pollen with bamboo chips and collect it with oil paper, then store it in the refrigerator under using the "Pollen collection and storage" conditions described in Section Materials and methods to reach 4 degrees Celsius and high humidity. When pollinating in the field, take it out and complete the pollination within 36 h. Additionally, from Fig 2 shows that the yield of artificial pollination was more than three times higher than that of natural pollination, and the economic value of FA is extremely high, the values are up to approximately $0.15 of a single FA.

The reproductive biology of *Amomum* plants are similar with the large cardamom, which’s floral longevity played a central role. Since the flowering period was very short, ending after only 1–1.5 days, pollination must occur right after anthesis, which occurs at morning hours [19]. In our study we found that simple low-temperature storage can delay the decline of pollen vitality after 24 h and prolong the vitality of live pollen for at least 9 h (Fig 1D). The time for artificial pollination was doubled, meaning that the yields of FA increased by six-fold on average in artificial pollination compared to natural pollination. Thus, they have practical implications in terms of advice for farms. The exact mechanism of the pollen long time storage solutions still needs to be explored in the future. In addition, although artificial pollination is currently the most effective method, artificial pollination requires heavy physical labor. Therefore, exploring the development of new varieties through the biological characteristics of multiple germplasms are still the focus of future research.

## Conclusion

This study has brought a new insight in improving the yield of *Amomum villosum*, indicating that the low yield is the result of a combination of factors, mainly due to the special flower structure, pollen viability and stigma pollination time is not long, effective pollination insects are few and climate warming causes early flowering that is out of sync with insect hatching. It is confirmed that late-flowering varieties are contribute to increased fruit set of *Amomum villosum* resulting into high yield, but artificial pollination is still the most effective method to increase yield. Compared to the in vitro germination test of *Amomum* plants, the TTC staining method is simpler and more accurate. Meanwhile, Simple low-temperature storage can delay the decline of pollen vitality after 24 h and prolong the vitality of live pollen for at least 9 h, which provides more time for artificial pollination by farmers over the entire day and is of great significance for field production.

## Supporting information

S1 Fig Schematic diagram about pollination. (TIF)

S2 Fig Results of verification by hybridization. (A) ‘*A*. *villosum cv*. *Changguo*’ (♀), natural-pollination. (B) ‘*Amomum longiligulare*’ (♂) ×‘*A*. *villosum cv*. *Changguo*’(♀), the pollen of ‘Amomum longiligulare’ was taken from storage at 4°C and high humidity after approximately 30 hours. (C) ‘*Amomum longiligulare*’ (♂) ‘*A*. *villosum cv*.*Changguo*’(♀), hand pollinated during a flowering season.
(EPS)

S3 Fig Number of florets on each inflorescence. (TIF)
