# Supplementary figures and images for: Research on the reproductive biological characteristics of Amomum villosum Lour. and Amomum longiligulare T. L. Wu
Source: PLoS One. 2021 Aug 19;16(8):e0250335. doi: 10.1371/journal.pone.0250335 (PMC8376005; doi:10.1371/journal.pone.0250335)

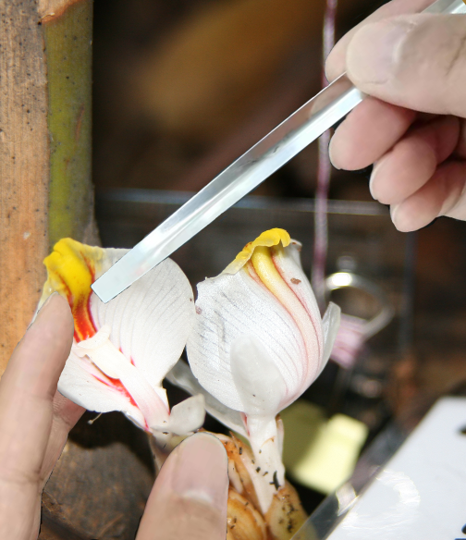

Supplement: S1 Fig — (TIF) [file pone.0250335.s001.tif]

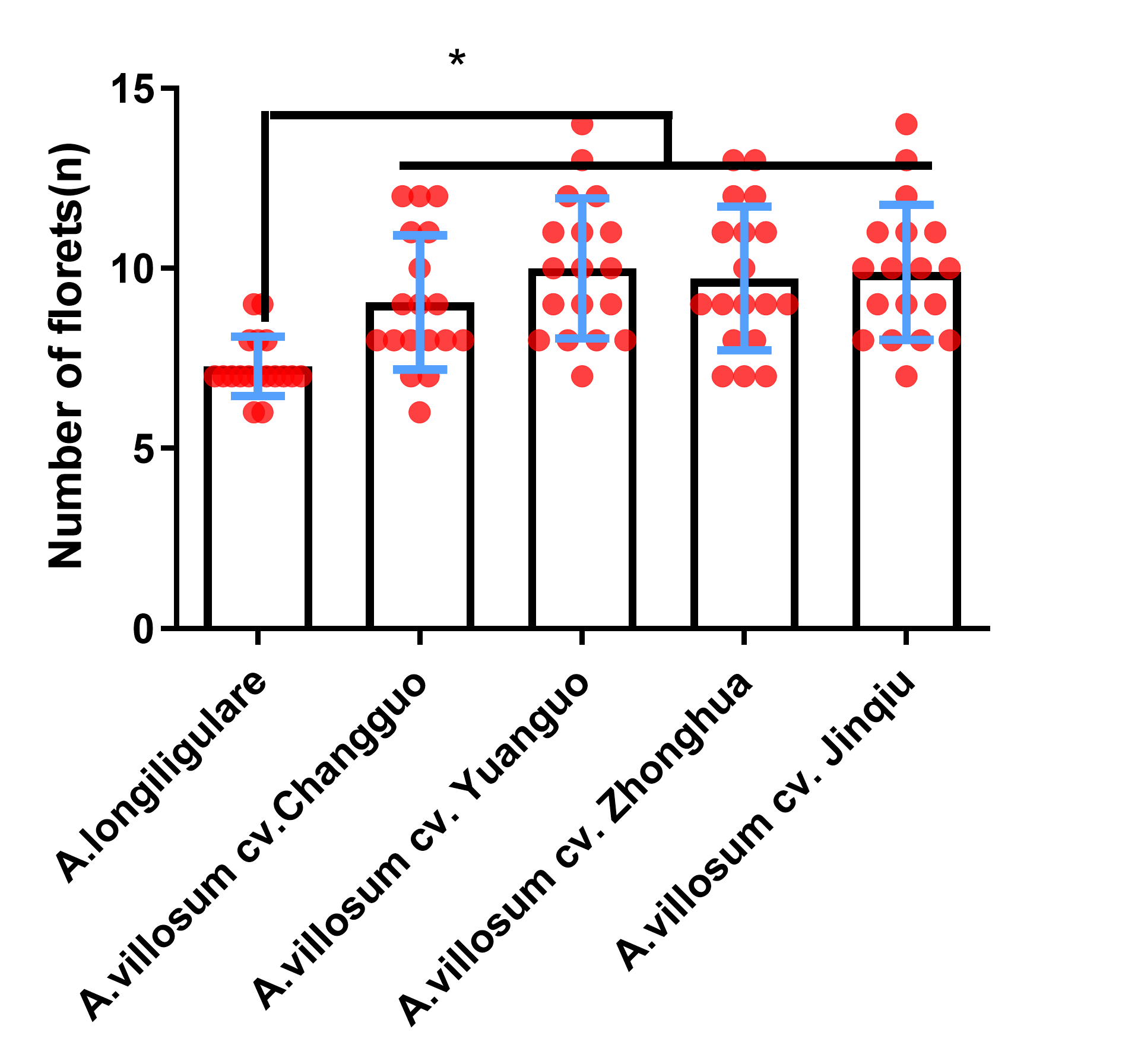

Supplement: S3 Fig — (TIF) [file pone.0250335.s003.tif]
